# Phenotype Profiling and Allergy in Otitis-Prone Children

**DOI:** 10.3389/fped.2018.00383

**Published:** 2018-12-04

**Authors:** Sara Torretta, Lorenzo Pignataro, Daniela Carioli, Tullio Ibba, Francesco Folino, Chiara Rosazza, Miriam Fattizzo, Paola Marchisio

**Affiliations:** ^1^Fondazione IRCCS Ca' Granda Ospedale Maggiore Policlinico, Milan, Italy; ^2^Department of Clinical Sciences and Community Health, University of Milan, Milan, Italy; ^3^Department of Pathophysiology and Transplantation, University of Milan, Milan, Italy

**Keywords:** acute otitis media, allergy, children, otitis media with effusion, atopy

## Abstract

**Background:** Otitis-prone children can present some distinctive clinical patterns and although a number of known risk factors for recurrent acute otitis media (RAOM) are known, no dedicated epidemiological models have been developed to explain clinical heterogeneity.

**Methods:** A preliminary retrospective pilot study was planned to evaluate the possible effect of allergic disease in the development of different disease phenotypes in otitis-prone children aged 3–10 years, particularly the absence (simple RAOM), or presence of episodes of otitis media with effusion between acute infections (RAOM with OME).

**Results:** Analysis was based on the data contained in 153 charts (55.6% males, mean age of 59.4 ± 16.4 months). 75.8% of children had a simple RAOM and 24.2% a RAOM with OME. Atopy or allergy were documented in respectively 47.7 and 41.3% of children considered as a whole. The prevalence of atopy or allergy was significantly higher in the children with a RAOM with OME (atopy: 73.0 vs. 39.5%, *p* < 0.001; allergy: 60.0 vs. 36.1%, *p* = 0.049), who also more frequently showed adenoidal hypertrophy (*p* = 0.016), chronic adenoiditis (*p* = 0.007), conductive hearing loss (*p* = 0.004), and impaired tympanometry (*p* < 0.001).

**Conclusions:** These data suggest that children with a RAOM with OME are clinically different from children with simple RAOM, as they have a more complex clinical presentation that includes not only adenoidal disease and audiological impairment, but also an underlying allergy or atopy. The possibility that the factors mentioned above may be differently involved in the heterogeneous clinical manifestations occurring in otitis-prone children needs to be further investigated in *ad hoc* epidemiological studies.

## Introduction

Recurrent and chronic middle ear diseases are frequently encountered in otorhinolaryngological and pediatric clinical practice, and they often require specialist consultations and antibiotic prescriptions (1–3). The most frequent of these is acute otitis media (AOM), which may also be the most frequent bacterial disease affecting infants and young children (4) as almost all children have experienced at least one episode by the age of 3 years, and about one-third have experienced three or more episodes (5). In addition, it has been estimated that up to 90% of children experience least one episode of otitis media with effusion (OME, defined as the presence of middle ear fluid for more than 3 months without any sign or symptom of ear infection) (6) before reaching school age, with most cases occurring by the age of 2 years (7–9). at Finally, some children are predisposed to recurrent AOM (RAOM: i.e., at least three documented and distinct AOM episodes in the preceding 6 months, or at least four in the preceding 12 months) (10), whose negative medical, social and economic effects can considerably worsen their and their parents' quality of life. In addition, some patients may develop complicated disease with possible severe and sometimes life-threatening events requiring urgent management such as mastoiditis, meningitis, and cerebral abscess.

Although the different clinical manifestations of recurrent or chronic middle ear inflammation, including RAOM and OME, have distinctive clinical and histopathological features, they sometimes overlap and are generally considered as belonging to a continuum of diseases affecting the so-called “otitis-prone” children (11). However, on the basis of our clinical experience and as previous described (12), we believe that it is possible to identify some distinctive patterns within this nosological entity.

There are a number of known risk factors for the development of chronic or recurrent middle ear inflammation, such as a genetic predisposition, a young age, male gender, little or no breastfeeding, parental smoking, the use of a pacifier or push-and-pull plastic bottle caps, day-care attendance, allergy, nasopharyngeal bacterial biofilm, and the presence of an older sibling (2, 13–18), but no dedicated epidemiological models have been developed to explain the clinical heterogeneity of this subset of diseases, and there is no unanimous consensus about the best individual approach to the single phenotypes. Moreover, although various interventions have been proposed as a means of reducing the risk of new infectious exacerbations in otitis-prone children (2, 3, 19–22), none of these is completely effective, and their benefits in terms of preventing RAOM have not been precisely quantified (2). We speculate that this may be related to the fact that different risk factors (or combinations of risk factors) can give rise to different clinical phenotypes that require different prophylactic interventions.

The aim of this preliminary retrospective pilot study (the first of a planned series of studies designed to investigate whether traditional risk factors differently influence particular disease patterns) was to evaluating the possible effect of allergic disease in the development of different disease phenotypes in otitis-prone children, particularly the presence of episodes of OME between acute infections, and the presence of spontaneous tympanic membrane perforations during episodes of AOM.

## Materials and Methods

### Study Design and Setting

This retrospective chart review of prospectively recruited otitis-prone children was carried out at Milan University's Department of Clinical Sciences and Community Health and Department of Pathophysiology and Transplantation in September 2017.

The protocol was approved by our local Ethics Committee of Fondazione IRCCS Ca' Granda Ospedale Maggiore Policlinico and was conducted in accordance with the principles of good clinical practice.

### Study Subjects

The study involved the charts of children aged 3–10 years who had attended our tertiary outpatient clinic of upper respiratory tract infection between November 2016 and May 2017. The children had been referred to the clinic by the pediatricians who had dealt with their initial episodes of AOM but were unable to manage recurrences effectively.

The children were considered otitis-prone if they had a history of RAOM with or without OME between AOM episodes (11). The episodes of AOM had to be documented by an experienced physician and reported in the children's medical records as any combination of fever, earache, irritability, and hyperemia or opacity accompanied by bulging of the tympanic membrane or otorrhea; at least two episodes had to be supported by otoscopic and tympanometric findings. RAOM was defined as at least three episodes in the preceding 6 months, or at least four episodes in the preceding 12 months (10), and OME as the presence of middle ear effusion for at least 3 months without any signs of concomitant acute middle ear inflammation, documented by means of pneumatic otoscopy and tympanometry (6).

The exclusion criteria were concomitant systemic diseases; craniofacial, neuromuscular, immunological, syndromic, or defined genetic abnormalities; chronic eardrum perforation; previous ear surgery (including ventilation tube placement) or adenoidectomy; neurosensory hearing loss; immunomodulatory treatment; vitamin D supplementation; the administration of any complementary and alternative medicines; and clinical signs or a history of suspected laryngo-pharyngeal reflux.

### Interventions

The retrospective chart review considered the children's demographic data and clinical history, with particular reference to the presence of allergy or atopy and their phenotypic pattern of disease (12). Allergy had to be documented by a positive skin prick or radio-allergosorbent test, and atopy by increased serum IgE levels within the previous 12 months with concomitant symptoms of asthma and\or rhinocongiuntivitis, and\or eczema. The children with RAOM but no OME between episodes were classified as having a simple RAOM, and those with RAOM and episodes of OME between acute exacerbations as RAOM with OME. Children were clinically evaluated during acute episodes and then 7 days, 1 month, and 3 months later with pneumatic otoscopy and tympanometry. The group RAOM with OME included only children without any evidence of complete clearance of the middle ear from effusion at any of the follow-up visits, documented by impaired findings at tympanometry, i.e., children with evidence at pneumatic otoscopy and/or tympanometry of middle ear effusion persisting for at least 3 months without any signs of concomitant acute middle ear inflammation.

The presence of recurrent spontaneous tympanic membrane perforation (RSTMP), defined as purulent ear discharge and/or otoscopic detection of tympanic membrane perforation occurring during ≥75% of AOM episodes (12), was also recorded.

The other data considered were age at the time of onset and at the time of satisfying the diagnostic criteria for RAOM; the number of AOM episodes since birth and in the previous 3, 6 and 12 months; the presence of hypertrophic adenoids or chronic adenoiditis revealed by flexible nasopharyngeal fiberendoscopy (23), and the results of an audiometry test (i.e., pure tone or conditioned play audiometry and tympanometry) carried as previously described (24). Hypertrophic adenoids were defined as the presence of Cassano grade ≥3 adenoidal pads (25), and chronic adenoiditis as the presence of hypertrophic adenoids with ongoing nasopharyngeal inflammations or infections (at least three episodes of acute adenoiditis requiring antibiotic therapy in a period of 6 months, or at least four episodes in a period of 12 months) (26).

Finally, a record was made of the factors known to influence the development of RAOM (including a family history of allergy, little or no breast-feeding, the use of a pacifier or push-and-pull plastic bottle caps, parental smoking, pneumococcal conjugate and influenza vaccination, day-care attendance, and the presence and number of older siblings) (2, 13, 16–18).

### Statistical Analysis

The sample size was computed considering published data regarding the prevalence of atopy in children with middle ear disease (27); assuming a standard deviation of 0.15, it was calculated that 34 subjects for each group would lead to a beta error margin of 0.20, an alpha value of 0.05, and a power of 80%.

The statistical analysis was mainly designed to detect possible association between the clinical phenotypic pattern of disease and the presence of allergy or atopy, and the effect of possible confounders on this relationship.

The results are given as absolute numbers and percentages, or arithmetical mean values ± standard deviation. Dichotomous outcomes were analyzed using contingency table analysis and Chi squared test, and continuous variables using Wilcoxon-Mann-Whitney test. Poisson's models for logistic multivariate regression analysis were used to assess the effect of possible confounders. The data were analyzed using STATA 10.0 software (StataCorp, College Station, TX, USA); a *p*-value of < 0.05 was considered statistically significant.

## Results

The final analysis was based on the data contained in 153 charts relating to 85 males (55.6%) and 68 females (44.4%) with a mean age of 59.4 ± 16.4 months. One hundred and sixteen children (75.8%) had a simple RAOM and 37 (24.2%) a RAOM with OME. Recurrent spontaneous tympanic membrane perforation occurred in 54 children (35.3%), and atopy or allergy were documented in respectively 73 (47.7%) and 38 (41.3%) (Table 1).

**Table 1:** Demographic and clinical characteristics of the study population as a whole and by the clinical phenotypic pattern of disease.

**Characteristics**	**Total (*n* = 153)**	**Simple RAOM (*n* = 116 pts)**	**RAOM with OME (*n* = 37)**	***p*-value**
Mean age ± SD, months	59.4 ± 16.4	59.0 ± 1.5	60.6 ± 2.8	n.s.
Males	85 (55.6%)	69 (59.5%)	16 (43.24%)	n.s.
RSTMP	54 (35.3%)	43 (37.1%)	11 (29.7)	n.s.
Increased IgE levels	73 (47.7%)	46 (39.7%)	27 (73.0%)	<0.001
Allergy	38 (41.3%)	26 (36.1%)	12 (60.0%)	0.049
Mean age at onset ± SD, months	20.2 ± 13.3	20.7 ± 1.5	19.7 ± 1.5	n.s.
Mean age at diagnosis ± SD, months	31.8 ± 16.0	30.3 ± 2.2	36.2 ± 2.8	n.s.
Mean No. of AOMs since birth ± SD	9.6 ± 4.6	9.4 ± 0.5	9.7 ± 0.5	n.s.
Mean No. of AOMs in previous 3 months ± SD	1.6 ± 0.8	1.4 ± 0.6	1.7 ± 0.5	n.s.
Mean No. of AOMs in previous 6 months ± SD	3.6 ± 1.6	3.4 ± 1.2	3.6 ± 1.8	n.s.
Mean No. of AOMs in previous 12 months ± SD	4.5 ± 2.1	4.3 ± 2.0	4.6 ± 2.4	n.s.
Adenoidal hypertrophy	68 (44.4%)	41 (35.3%)	27 (73.0%)	0.016
Chronic adenoiditis	52 (34.0%)	31 (26.7%)	21 (80.8%)	0.007
Conductive hearing loss (ad endo)	35 (22.9%)	20 (17.2%)	15 (40.5%)	0.004
Impaired tympanometry^*^	79 (51.6%)	49 (42.2%)	30 (81.1%)	<0.001
Family history of allergy	58 (37.9%)	44 (37.9%)	14 (37.8%)	n.s.
Prematurity	14 (9.1%)	10 (8.6%)	4 (10.8%)	n.s.
Little or no breastfeeding	57 (37.2%)	44 (37.9%)	13 (35.1%)	n.s.
Day-care attendance (%)	149 (96.7%)	11 (95.7%)	37 (100%)	n.s.
Use of pacifier	61 (39.9%)	50 (43.1%)	11 (29.7%)	n.s.
Use of push-and-pull plastic bottle caps use	66 (43.1%)	54 (46.5%)	12 (32.4%)	n.s.
Parental smoking	68 (44.4%)	55 (47.4%)	13 (35.1%)	n.s.
Presence of older siblings	65 (42.5%)	48 (41.4%)	17 (45.9%)	n.s.
Mean No. of siblings ± SD	1.5 ± 0.7	1.5 ± 0.1	1.4 ± 0.1	n.s.
PVC7 immunization	136 (88.9%)	101 (87.1%)	35 (94.6%)	n.s.
Influenza immunisation	60 (39.5%)	43 (37.4%)	17 (45.9%)	n.s.

SD, standard deviation; RAOM, recurrent acute otitis media; OME, otitis media with effusion; n.s., not significant; RSTMP, recurrent spontaneous tympanic membrane perforation; AOM, acute otitis media; PVC7, pneumococcal conjugate vaccine;

* *Type B or C-tympanogram in at least one ear*.

The prevalence of atopy or allergy was significantly higher in the children with a RAOM with OME (atopy: 73.0 vs. 39.5%, *p* < 0.001; allergy: 60.0 vs. 36.1%, *p* = 0.049), who also more frequently showed adenoidal hypertrophy (*p* = 0.016), chronic adenoiditis (*p* = 0.007), conductive hearing loss (*p* = 0.004), and impaired tympanometry (*p* < 0.001). The other main demographic and clinical characteristics were comparable in the two groups (Table 1). These factors were confirmed to be independent predictors at logistic multivariate regression analysis (Table 2).

**Table 2:** Results of multivariate logistic regression models.

**Variable**	***P*-value**	**Pseudo *R*^2^**
Increased IgE level	< 0.001	0.57
Allergy	< 0.001	0.60
Adenoidal hypertrophy	0.003	0.53
Chronic adenoiditis	0.002	0.52

There was no statistically significant association between the presence of allergy or atopy and chronic adenoiditis or adenoidal hypertrophy: chronic adenoiditis affected respectively 40.0 and 40.7% of the allergic and non-allergic children, and 43.3 and 48.6% of the atopic and non-atopic children; adenoidal hypertrophy was detected in 51.7 and 30.8% of the allergic and non-allergic children, and in 42.2 and 47.2% of the atopic and non-atopic children.

Conductive hearing loss was more frequently detected among the children with allergy (39.5 vs. 9.4%, *p* = 0.001), but there was no significant difference between atopic and non-atopic children (26.0 vs. 20.0%).

The children with allergy satisfied the diagnostic criteria for RAOM slightly but not significantly later than the non-allergic children (36.2 ± 2.8 vs. 30.3 ± 2.2 months), and there was no difference between the atopic and non-atopic children (32.8 ± 1.9 vs. 30.8 ± 1.7 months).

There was no significant association between allergy or atopy and RSTMP, which occurred in respectively 51.8 and 48.1% of the atopic and non-atopic children, and in 43.2 and 56.8% of the allergic and non-allergic children.

## Discussion

This first study evaluating the role of allergic/atopic status in otitis-prone children with RAOM with (RAOM with OME) or without OME (simple RAOM) between episodes is part of the broader Epidemiology and Phenotypes In Children with Otitis (EPICO) project aimed at assessing the impact of the most frequent traditional and newly described risk factors for recurrent or chronic middle ear inflammation on phenotypes (12).

The importance of distinguishing otitis media phenotypes has previously been discussed by us (12) and Bhutta et al. (28) who has constructed sophisticated mathematical models to represent the clinical inter-relations between individual clinical manifestations and the impact of variables, such as upper respiratory tract infections in a new disease scenario.

The findings of this preliminary retrospective pilot study show that the prevalence of allergy or atopy patients was significantly higher in otitis-prone children with a RAOM with OME than in those with a simple RAOM: atopy was found in 73.0% of the children with a RAOM with OME as against 39.5% of those with a simple RAOM, and allergy was found in respectively 60.0 and 36%, which suggests that allergy may differentially account for different phenotypic pattern of disease in children with RAOM, and may be involved in the development of a RAOM with OME.

Allergy and atopy have been previously investigated as possible risk factors for AOM and OME (18, 29–37). However, although there is clinical, mechanical and therapeutic evidence supporting their possible effect on the pathogenesis of recurrent or chronic middle ear inflammation, there is still a lack of strong epidemiological evidence, and the results obtained in previously published studies are conflicting. One meta-analysis (29) published in 1996 pooled the results of three studies (30–32) involving a total of 1,362 children failed to find any statistically significant impact of atopy or allergy on AOM (relative risk 1.23; 95% confidence interval 0.94–1.61), and the same is true of the studies by Pukander et al. (33) and Staaberg et al. (34). However, Kvaerner et al. (35) used a cross-sectional questionnaire to investigate 40,000 randomly selected Norwegian adults and found that surgery for RAOM and otitis media was more frequent in the respondents with allergy, and Kramer et al. (36) found that atopic symptoms were approximately four times more frequent in children with OME who were candidates for tympanostomy tube placement than in control children undergoing general pediatric surgery. Beside this, some evidences support the hypothesis that OME should be enumerated among allergic manifestations, considering that middle ear mucosa has been found to have the same active intrinsic immunologic responsiveness to antigens as the upper and lower respiratory ectodermal-derived tract epithelium (38). Moreover, it has been hypothesized that allergic inflammation into the middle ear during OME would depend not only on activation of secretory immunity following direct allergen transport, but also on humoral and cell-mediated reaction (39).

We also found that adenoidal disease (adenoidal hypertrophy or chronic adenoiditis) was significantly more prevalent among children with RAOM with OME than among those with a simple RAOM (adenoidal hypertrophy: 73.0 vs. 35.3%; chronic adenoiditis: 80.8 vs. 26.7%). However, as there was no significant association between the presence of atopy/allergy and adenoidal disease, it can be assumed that allergic status was not directly related to the development of nasopharyngeal disease in our children. The possible causative role of adenoidal disease in otitis-prone children has been previously postulated by Salah et al. (40) who identified adenoidal hypertrophy as a possible risk factor for treatment failure and infectious recurrences in children with RAOM in a retrospective study of 340 otitis-prone infants.

Our findings confirm that children with a RAOM with OME more frequently have impaired hearing than children with simple RAOM, and show that they satisfy the diagnostic criteria for RAOM slightly later, at a mean age of about 36 months. The latter finding is particularly interesting because, as most of the clinical manifestations of allergy in atopic children appear after the age of 3 years (41), we are tempted to hypothesize that a RAOM with OME could be such a manifestation, which is why we only recruited children aged >3 years.

Taken together, these data suggest that children with a RAOM with OME are clinically different from children with simple RAOM, as they have a more complex clinical presentation that includes not only adenoidal disease and audiological impairment, which may pre-dispose them to long-term functional sequelae and become a major cause of surgical treatment, but also an underlying allergy or atopy requiring second-level assessment and anti-allergic therapy. However, the possible causative role of an allergic status (or adenoidal disease) in the pathogenesis of RAOM with OME goes beyond the scope of this preliminary retrospective study, and it should be addressed by *ad hoc* epidemiological studies.

Possible limitations of the present study lie in its retrospective nature which may partially bias our results, and in the reduced sample size; therefore, prospective controlled trials would be welcome to confirm our findings.

The possibility that the factors mentioned above may be differently involved in the heterogeneous clinical manifestations occurring in otitis-prone children needs to be further investigated in *ad hoc* epidemiological studies. If our theory is confirmed, it would have important clinical and therapeutic consequences as it would allow dedicated diagnostic, therapeutic and prophylactic interventions to be designed for each clinical pattern.

**Figure 1:**
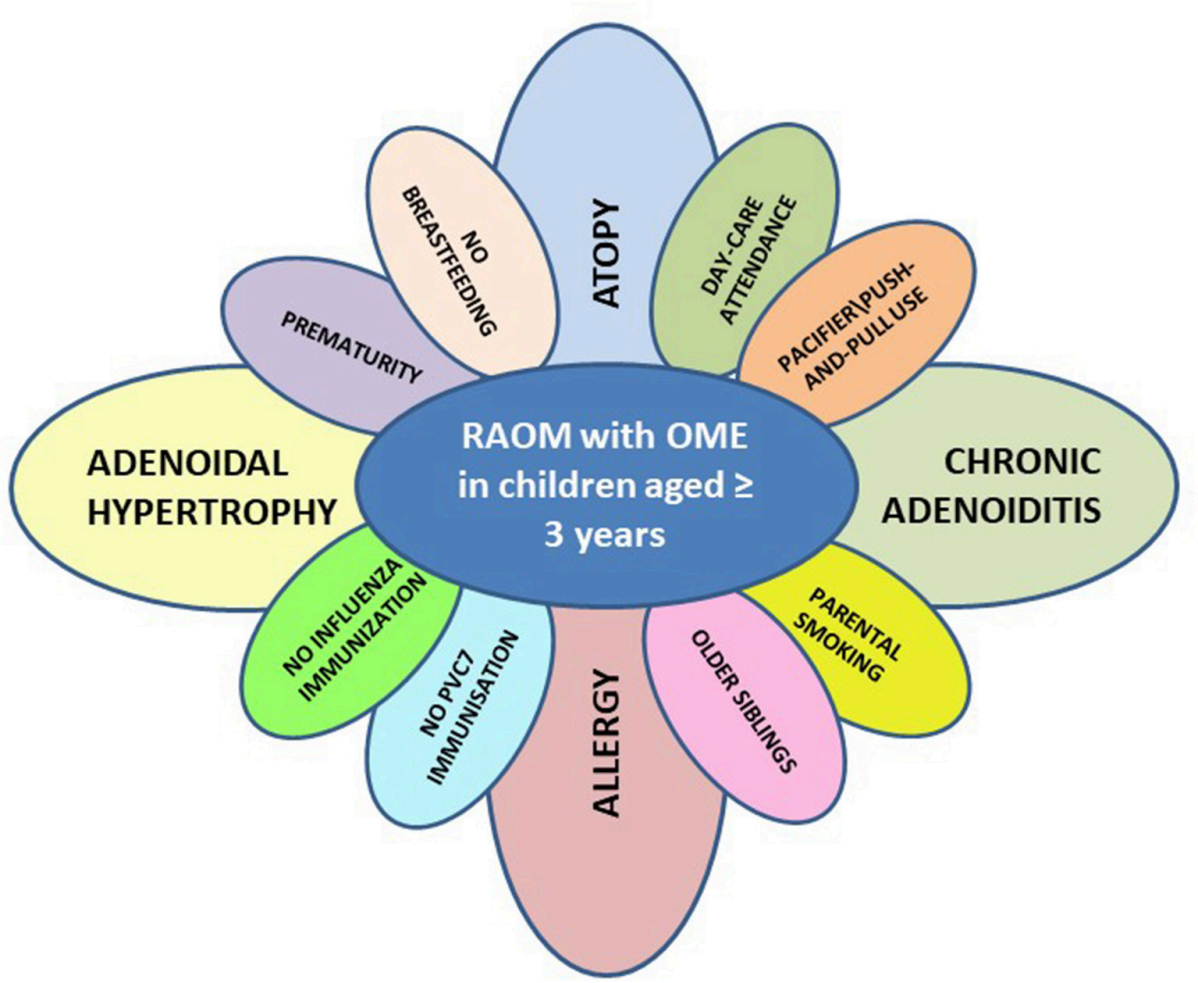
Risk factors for phenotypic pattern of disease characterized by the presence or recurrent acute otitis media with persisting otitis media with effusions among acute episodes (RAOM with OME) in children aged ≥3 years (PVC7, pneumococcal conjugate vaccination).

Finally, the nosological and epidemiological concepts underlying our view of childhood recurrent and chronic middle ear disease could be updated, and the famous illustration of the risk factors for pediatric RAOM known as Rovers' daisy (42) could ideally be reproduced in serial copies for each phenotypic pattern of disease and different ages with the petals' size being weighted on the basis of their etiological relevance, as we have tried to do in the case of children with RAOM with OME aged >3 years (see Figure 1).

## Author Contributions

ST drafted the manuscript. PM helped in drafting the manuscript. FF, MF, and CR performed data extraction. TI and DC performed literature search. LP made important intellectual contributions to the paper.

### Conflict of Interest Statement

The authors declare that the research was conducted in the absence of any commercial or financial relationships that could be construed as a potential conflict of interest.
